# A Novel Hybrid Secure Image Encryption Based on the Shuffle Algorithm and the Hidden Attractor Chaos System

**DOI:** 10.3390/e22060640

**Published:** 2020-06-09

**Authors:** Xin Jin, Xintao Duan, Hang Jin, Yuanyuan Ma

**Affiliations:** 1 College of Computer and Information Engineering, Henan Normal University, Xinxiang 453007, China; jx18336066598@gmail.com (X.J.); 121100@htu.edu.cn (Y.M.); 2 Economics and Management School, Wuhan University, Wuhan 430072, China; jinhang@whu.edu.cn

**Keywords:** security analysis, image encryption, shuffle algorithm, chaotic system, DNA sequence

## Abstract

Aiming at the problems of small key space, low security of encryption structure, and easy to crack existing image encryption algorithms combining chaotic system and DNA sequence, this paper proposes an image encryption algorithm based on a hidden attractor chaotic system and shuffling algorithm. Firstly, the chaotic sequence generated by the hidden attractor chaotic system is used to encrypt the image. The shuffling algorithm is used to scramble the image, and finally, the DNA sequence operation is used to diffuse the pixel value of the image. Experimental results show that the key space of the scheme reaches 2^327^ and is very sensitive to keys. The histogram of encrypted images is evenly distributed. The correlation coefficient of adjacent pixels is close to 0. The entropy values of encrypted images are all close to eight and the unified average change intensity (UACI) value and number of pixel changing rate (NPCR) value are close to ideal values. All-white and all-black image experiments meet the requirements. Experimental results show that the encryption scheme in this paper can effectively resist exhaustive attacks, statistical attacks, differential cryptanalysis, known plaintext and selected plaintext attacks, and noise attacks. The above research results show that the system has better encryption performance, and the proposed scheme is useful and practical in communication and can be applied to the field of image encryption.

## 1. Introduction

In recent years, the wide application of the internet and the popularization of digital information have brought significant changes to people’s life and learning styles [1]. Digital information has been widely used in online teaching, medical imaging, secure communication, and other fields due to its characteristics of easy access, convenient replication, and rapid dissemination, which greatly enriches people’s lives. The digital image is a form of digital information and has been widely used. However, using the openness and sharing of the network to intercept information has seriously harmed the interests of all parties in communication. Therefore, it is increasingly important to design practical and reliable encryption schemes. The encryption of digital images has received extensive attention. 

Digital images are characterized by strong pixel correlation, large capacity, and high redundancy. Therefore, the computationally intensive and time-consuming DES and AES algorithms [2,3] are not suitable for real-time image transmission. Thus, technologies based on chaotic systems [4,5,6,7,8,9,10,11,12,13,14,15], deoxyribonucleic acid (DNA) sequences [14,15], quantum walk [16,17,18,19], cellular automata [20,21], and the like are widely applied to image encryption algorithms [22]. On the other hand, chaotic systems are especially suitable for image encryption because of their sensitivity to initial conditions and control parameters, the density of periodic points, and topological transitivity. In recent years, a large number of image encryption algorithms based on chaos have been proposed one after the other [4,5,6,7,8,9,10,11,12,13,14,15]. Yin Q et al. proposed a more sensitive chaotic image encryption scheme based on permutation and diffusion structure, using breadth-first search and dynamic diffusion to enhance security and sensitivity [13]. Chai Xiuli et al. used a hyperchaotic memory system, cellular automata, and DNA sequence operation to encrypt images [15]. Self-excited attractors generate chaotic systems in general encryption algorithms. The attraction domain of the self-excited attractor is related to the equilibrium point. Common chaotic systems can be restored by reconstructing the attractor, which will significantly reduce the security of the encryption algorithm [23].

In contrast, the attraction domain of hidden attractor chaotic systems does not intersect with any neighborhood of the equilibrium point. Attackers cannot restore the chaotic system by reconstructing the attractor, and the security is much better than that of ordinary chaotic systems [23,24,25,26,27,28,29,30]. Due to less research on hidden attractor chaotic system, hidden attractor chaotic system is seldom applied to the image encryption scheme. Cavusoglu et al. [31] focused on the process of generating the hidden attractor chaotic system and did not introduce too much the effect of image encryption. In this paper, a large number of experiments are carried out to fully demonstrate the encryption effect of the encryption algorithm based on the hidden attractor chaotic system, and the security and performance of the algorithm are analyzed.

To resist attackers’ statistical attacks on the encryption system, C.E. Shannon proposed two necessary steps for designing the encryption system: diffusion and confusion. Hence, the image encryption algorithm usually includes these two steps. Many researchers have proposed many practical permutation algorithms such as Arnold transform, Hilbert curve, and baker transform [32,33,34,35]. However, these classical methods also have many problems, such as obvious periodicity and weak randomness. Shuffle algorithm has good randomness, but Erdal Guvenoglu et al. applied the Knuth–Durstenfeld algorithm to generate keys instead of confusion [36]. In this paper, the performance of the algorithm is analyzed through a large number of experiments, and the experimental results sufficiently prove that while guaranteeing the image scrambling effect, the Knuth–Durstenfeld shuffling algorithm can reduce the time complexity and space complexity of the algorithm.

Based on the above analysis, this paper proposes a novel hybrid secure image encryption based on a shuffling algorithm and hidden attractor chaotic system. The advantages of the algorithm proposed in this paper are as follows:

1. The hidden attractor chaotic system is applied to image encryption. The hidden attractor chaotic system is easily affected by initial values and parameters, and the attacker cannot reconstruct the attractor to crack the chaotic system.
2. The Knuth–Durstenfeld shuffling algorithm is used in the shuffling process. Knuth–Durstenfeld algorithm has lower space complexity and time complexity.
3. In the encryption process, the key consists of the chaotic sequence of the chaotic system and the hash sequence of the image, which ensures the security of transmission.

The structure of the paper is as follows: The second section introduces the preliminary work and methods. The third section introduces the proposed image encryption scheme. The fourth section, experimental results, and analysis. The fifth section, safety, and performance analysis. The sixth section is the conclusion of the thesis.

## 2. Preliminary Work and Methods 

### 2.1. Chaotic System

In this paper, we generalized the chaotic system from the generalized non-diffusion Lorenz equation and obtained the four-dimensional implicit attractor hyperchaotic system. The system has the characteristics of double scroll chaos, periodic dynamics, and quasi-periodic.

#### 2.1.1. Hidden Attractor Hyperchaotic System

The system is a four-dimensional continuous chaotic system, and its equation is shown in formula (1):

(1) $$\left\{ \begin{array}{l} \dot{x}=a\cdot (y-x)\\ \dot{y}=-xz-cy-kh\\ \dot{z}=-b+xy\\ \dot{h}=-my \end{array}\right.$$

In the system (1), the system can show different characteristics under different conditions, as shown in Table 1. When the system is a hidden attractor chaotic system, are the real parameters of the system.

According to the hyperchaos theory, for a four-dimensional hyperchaos system, at least two Lyapunov exponents are positive. Different initial conditions and parameters will make the system in different states. Specific examples are shown in Table 1.

The tiny change of the initial state of the system results in the different trajectories, and the experiment takes parameter c as an example. The influence of parameter c on the system is shown in Table 2. Tests show that chaotic systems show different dynamic characteristics with the change of parameters.

**Figure 1:**
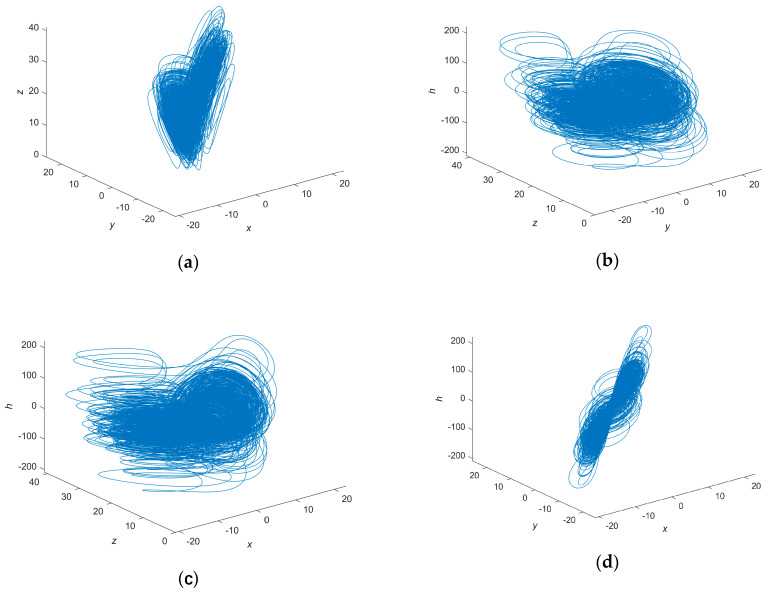
The hidden attractor hyperchaotic system. (**a**) *x*-*y*-*z*; (**b**) *y*-*z*-*h*; (**c**) *x*-*z*-*h*; (**d**) *x*-*y*-*h*.

Figure 1 shows the attractor phase diagram of a hidden attractor hyperchaotic system. The hidden attractor hyperchaotic system has no equilibrium point. There is no equilibrium point in the hyperchaotic system. The shape of the hidden hyperchaotic attractor is a double scroll, similar to the butterfly shape of the Chen hyperchaotic attractor.

#### 2.1.2. System Randomness Test

To prove that the sequence generated by the chaotic system meets the requirements of image encryption, fits the characteristics of random sequence, and meets the required random standard [37], according to the SP800-22 standard formulated by the National Institute of Standards (NIST) and TESTU01 statistical test suite, the output sequence of the implicit attractor chaotic system is tested for randomness. The output of the hidden attractor chaotic system in this paper adopts a double-precision data format, and its output is converted into a binary stream for testing. Table 3 shows the NIST test results. All *p*-values of the NIST test are evenly distributed in the interval, the average passing rate of the test is about 99.1%, and the passing rate is within the acceptable range. 

Due to the small amount of evaluation data used in NIST test standards, the required number of iterations of data evaluation is not large enough when chaotic sequences generated by chaotic systems are used for randomness detection, which will not expose the degradation of chaotic dynamics and achieve the purpose of effective testing. Therefore, the TESTU01 statistical characteristic test, which is stricter than the NIST test, will be adopted in this paper. The difference from NIST testing is that the amount of test data is larger, and the number of test items is more. The software library includes seven built-in module kits, namely, the primary test kit SmallCrush, the intermediate test kit Crush, the advanced test kit BigCrush, Alphabit, Rabbit, PseudoDIEHARD and FIPS-140-2. The specific testing method of TESTU01 is to test the generated chaotic sequence with the primary suite, then with the intermediate suite, then with the advanced suite, and finally with the four suites BigCrush, Alphabit, Rabbit, PseudoDIEHARD. The next test is necessary only if each test passes. The test results of chaotic sequences generated by the algorithm proposed in this paper are shown in Table 4 below, the chaotic sequence generated by the algorithm can pass the extremely strict TESTU01 test.

The test results show that the pseudo-random sequence generated by the hidden attractor chaotic system has successfully passed two tests and meets the requirements. Therefore, the hidden attractor chaotic system can be applied to image encryption.

### 2.2. Shuffle Algorithm

The shuffle algorithm includes drawing cards, changing cards, and insert cards. Shuffle algorithm is to break up the original array so that a certain number of the original array can appear with equal probability at each position in the broken array, wherein drawing cards and changing cards correspond to the Fisher–Yates Shuffle algorithm and the Knuth–Durstenfeld Shuffle algorithm, respectively [38].

#### 2.2.1. Fisher-Yates Shuffle Algorithm

The main steps of the Fisher–Yates shuffling algorithm are to randomly take a number that has not been made before from the original array to the new array. The algorithm has a time complexity of O(n × n) and a space complexity of O(n). The specific steps are as follows:

1. The length of the original array is known to be n, and the original array and the new array are initialized.
2. Assuming that there are still k arrays that have not been processed, and the value range of the array is [0, *k*], randomly generate a number P between the value ranges, and take out the value P from the array.
3. Repeat step 2 until all the numbers are taken and record them.
4. The number sequence recorded in step 3 is a scrambled number sequence.

#### 2.2.2. Knuth–Durstenfeld Shuffling Algorithm

The number has interacted on the original array of Fisher–Yates shuffling algorithm, which is Knuth–Durstenfeld shuffling algorithm. The time complexity and space complexity of the algorithm is reduced to O(*n*) and O(1), respectively. The specific steps are as follows:

1. Create a new array with a size of n, generate a random number *x*_1_ with a value range of [0, *n* − 1], and use *x*_1_ as the subscript of the random output value arr.
2. Exchange the suffix value of arr with the element of subscript *x_1_*.
3. Generate a random number *x_2_* with a value range of [0, *n* − 2], and use *x_2_* as the subscript of the output value arr, that is, the second random number.
4. Replace the penultimate value of arr with the element of subscript *x_2_*.
5. Process the array according to the rules of steps 1 to 4 until m values are generated.

### 2.3. DNA Sequence Operation

DNA (deoxyribonucleic acid) has the advantages of large-scale parallel, ample storage, and ultra-low power consumption. In recent years, it has been applied to the chaotic image encryption system [39].

#### 2.3.1. DNA Coding

DNA encoding is the process of binary mapping values to DNA bases. DNA composition contains four bases, of which A and T, C and G are complementary pairs, respectively. According to Watson Crick’s basic rule, 4! = 24, but only 8 of the 24 methods met the standard. Table 5 lists eight systems that satisfy the complementarity rule.

Suppose that the value of a pixel point of an image is decimal 114, the binary form is 01110010, DNA coding is carried out according to mode 1, sequence “CTAG” is obtained, the sequence is decoded according to mode 5, the binary number “00011011” is obtained, and conversion to decimal is “27”. It can be seen that through simple DNA encoding and decoding, a value can change significantly, making the digital image encryption effect better.

#### 2.3.2. DNA Algorithm

The DNA operation is based on the rule that every two binary values correspond to one DNA base. There are eight kinds of qualified DNA coding methods, and each method has a set of algorithms, so each commonly used algorithm corresponds to 8 different DNA algorithms. Use DNA exclusive OR (XOR) operation defined by DNA encoding mode 0 is shown in Table 6. If “ATGC” and “AGTC” are XOR, the result is “ACCA”.

## 3. The Proposed Encryption Scheme

The algorithm uses a chaotic system to generate a chaotic sequence and selects a chaotic sequence according to the hash value of the original image. Then the original image is scrambling by shuffling algorithm. Finally, the encrypted image is obtained by diffusing the image through DNA operation. The encryption algorithm in this paper not only encrypts the image safely but also ensures excellent encryption performance.

### 3.1. Encryption Process

**Figure 2:**
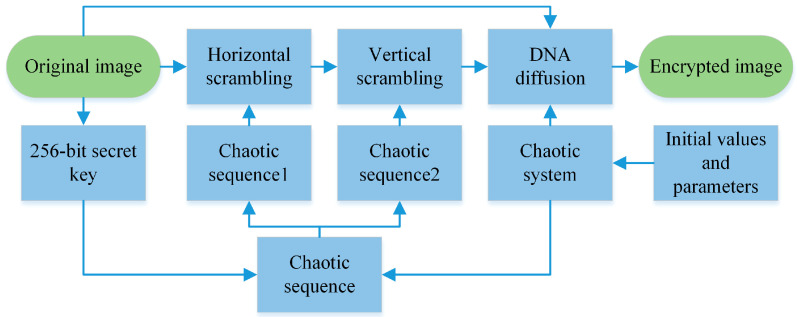
The encryption processes.

The process of the encryption algorithm proposed in this paper is shown in Figure 2.

Assuming the size of the original grayscale image P is M×N, the specific encryption process of the algorithm is as follows:

1. The hidden attractor chaotic system used in this paper is in double-scroll hyperchaos. The key of the algorithm consists of the hash value of the original image, the parameters and initial values of the chaotic system, wherein the parameters and initial values of the system are shown in the second row in Table 1.
2. To avoid the transient effect of the system, the chaotic system uses the key of step 1 to iterate 1000 times. To enhance the sensitivity of the encryption system, the generated chaotic sequence is divided into six different groups: $A_{1}(x,y),A_{2}(x,z),A_{3}(x,h),A_{4}(y,z),A_{5}(y,h),A_{6}(z,h)$.
3. Two variables hash and index are defined. According to the Secure Hash Algorithm 256 (SHA-256) algorithm, the hash value of the original image is obtained, and the hexadecimal hash value is converted into decimal number in turn and added to get the hash value. The specific method is shown in formula (2).
  (2) index=mod(hash,6)+1
  Mod (hash,6) indicates the remainder of the hash divided by 6. R1 and R2, respectively, represent vectors Ai(1) and Ai(2).
  (3) $$\begin{array}{l} When\;index=n\\ then\;A_{i}=A_{n},i=n\\ \left\{ \begin{array}{l} i=1,R_{1}=X,R_{2}=Y\\ i=2,R_{1}=X,R_{2}=Z\\ i=3,R_{1}=X,R_{2}=H\\ i=4,R_{1}=Y,R_{2}=Z\\ i=5,R_{1}=Y,R_{2}=H\\ i=6,R_{1}=Z,R_{2}=H \end{array}\right. \end{array}$$
  To achieve the scrambling effect, R1 and R2 are processed, as shown in Formula (4), and the processed results are set as vector Row and vector Column, respectively:
  (4) $$Vector(i)=\mathrm{mod}(floor((R_{n}(i)+100)\times 10^{10}),M\times N-i+1)+1,(n=1,2)$$
4. According to the shuffling algorithm, the chaotic sequence *R*_1_ processed in step 3 is used to scramble the original image, and the original image matrix *P* is modified into a one-dimensional vector *P*_Row. The scrambling process is shown in Formula (5).
  (5) $$P\_Row(R_{1}(i))=P\_Row(M\times N-i+1)$$
  The processed vector P_Row is transposed and expanded to obtain a one-dimensional vector P_Column.
5. According to formula (5), through the chaotic sequence *R_2_* pair *P*_Column to scramble. The processed sequence *P*_Column is re-converted into a matrix p of size *M×N*. P_1_ is calculated by the formula (6) to obtain the variable temp.
  (6) $$temp=\mathrm{mod}(\sum_{j=1}^{M\times N}P_{1},256)$$
6. The parameters and initial values of the chaotic system are set to the values in step 1, and the parameters and initial values of the chaotic system are iterated 1000 + *MN* times, thus avoiding the transient effect of the chaotic system, and their values are stored in the initial value sequences of the chaotic system, which are chaotic.
7. Through the formula (7) pair of four chaotic sequences, each element operates to obtain four-vectors *R_x_*, *R_y_*, *R_z_*, and *R_h_*.
  (7) $$\left\{ \begin{array}{l} R_{x}(i)=\mathrm{mod}(X1(i)’10^{10},8)+1\\ R_{y}(i)=\mathrm{mod}(Y1(i)’10^{10},8)+1\\ R_{z}(i)=\mathrm{mod}(Z1(i)’10^{10},8)+1\\ R_{h}(i)=\mathrm{mod}(H1(i)’10^{10},256) \end{array}\right.$$
  i represents the i-th element of four chaotic sequences, i∈[1,M×N], the matrix P1 is converted into a one-dimensional vector E(i).
8. According to the coding rules of *R_z_(i)* and *R_y_(i)*, *R(i)*, and *E(i)* are respectively DNA coded to obtain DR(*i*) and DE(*i*), NE(*i*) is obtained by XOR of DR(*i*) and DE(*i*).
9. According to the rules corresponding to *R_x_(i)*, *NE(i)* is decoded to obtain DNE(*i*). CNE(*i*) is obtained by XOR of DNE(*i*) and temp.
10. Loop through steps 8 and 9 until all elements of the original image are encrypted. Then the vector is transformed into a M×N matrix to obtain an encrypted image.

### 3.2. Decryption Process

The decryption process is the inverse of the encryption process. Before decrypting the image, the same key as the encryption process must be used. Since the image is in the hiding stage, the decryption sequence is generated in the same way as the encryption phase. Then decrypt the diffusion step, and finally decrypt the replacement step to decrypt the image.

**Figure 3:**
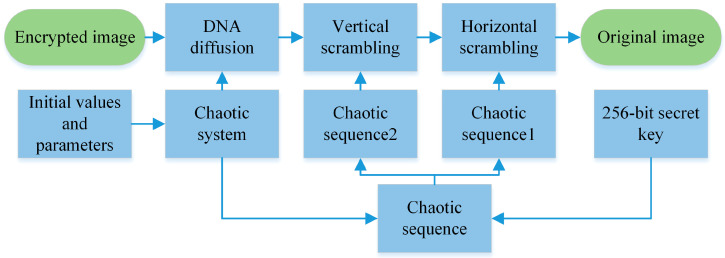
The decryption processes.

The process of the decryption algorithm proposed in this paper is shown in Figure 3. 

## 4. Experimental Results and Analysis

**Figure 4:**
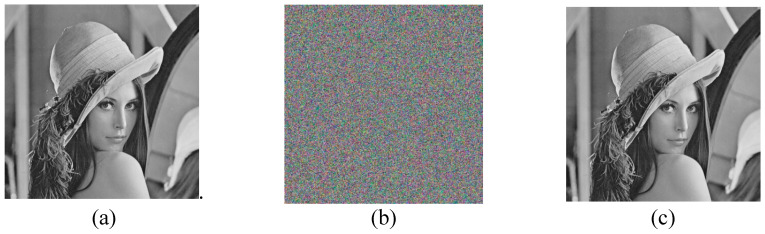
Encryption and decryption effect; (**a**) Original image of Lena; (**b**) Encrypted image of Lena; and (**c**) Decrypted image of Lena.

To verify the algorithm proposed in this paper, using MATLAB R2018a software simulates the algorithm. The primary hardware environment of the experimental equipment is the processor: Intel Core i5-6300HQ CPU @ 2.30 GHz; Installation memory: 8 GB; Operating system: Windows10 home Chinese version. The experimental parameters of the “encryption and decryption process” are shown in Table 7. The “Lena” standard 512 × 512 image is taken as an example, as shown in Figure 4.

The experimental results are shown in Figure 4. By looking at Figure 4b, it can be seen that the encrypted image is an unordered image without displaying any clear text information. There is no relationship between the original image and the encrypted image. The structural similarity between the original image and the encrypted image is analyzed, and the similarity is 1. The results show that the encryption and decryption effect of the algorithm is good.

## 5. Security Analysis

In the fifth section, the security of the encryption algorithm is evaluated through key space analysis, key sensitivity analysis, histogram analysis, correlation coefficient analysis of adjacent pixels, information entropy analysis, differential cryptanalysis analysis, noise attack analysis, known plaintext, and selective plaintext attack analysis.

### 5.1. Key Space Analysis

According to references, the key space of the algorithm must be large enough to resist various violent attacks, and the key space should be at least 2^100^ ≈ 10^30^ [40,41]. In this algorithm, the key space consists of three parts: SHA-256 function value of the original image, the initial values of the chaotic system, and parameters of a chaotic system. The ideal key space of SHA-256 is 2^128^, if the accuracy of four parameters *x*_0_, *y*_0_, *z*_0_, and *h*_0_ of a chaotic system is set to 10^−15^, the key space can reach 10^60^, and the key space of the algorithm can reach 2^327^, much larger than 2^100^ [42], Table 8 shows the key space comparison between the algorithm in this paper, and that in reference [43,44,45,46,47], the algorithm in this paper has a larger key space, it is generally believed that the algorithm’s key length up to 128 bit is secure. The algorithm’s key space has reached a security standard. From literature [48], it can be seen that the algorithm in this paper can resist all kinds of exhaustive attacks.

### 5.2. Key Sensitivity Analysis

A good encryption algorithm should be susceptible to keys. In the process of decrypting encrypted images, minor changes in the key will also cause the recovery of encrypted images to fail. The sensitivity of the algorithm key is analyzed to verify the security of the encryption algorithm. In the experiment, *x*_0_ in the original key is modified to *x*_0_ + 10^−15^, and uses the modified key set to decrypt the encrypted image.

In Table 9, the first column is the original image, the second column is the encrypted image, the third column is the decrypted image using the wrong key, and the fourth column is the decrypted image using the correct key. When the error rate of a single key reaches the order of 10^−15^, the original image cannot be obtained. When any one of the multiple keys is changed, the original image cannot be decrypted, as shown in Table 10. Experimental results show that the encryption algorithm is highly sensitive to keys.

### 5.3. Statistical Attack Analysis

#### 5.3.1. Histogram Analysis

The encryption system must make the encrypted image have a uniform histogram to resist statistical attacks because the image histogram represents the distribution of pixel intensity values in the image. Histograms of the original image and encrypted image are shown in Table 11. The abscissa of the histogram is the gray level, and the ordinate is the frequency of occurrence of the gray level.

The experimental results show that the pixels of the encrypted image obey the uniform distribution, that is to say, the frequency of each pixel value after encryption is very close, and the attacker will not be able to obtain the statistical law of the encrypted image. To verify whether the histogram of the encrypted image obeys uniform distribution, the encrypted image is quantized by chi-square test, and the formula is as shown [49];

(8) $$\chi 2=\sum_{i=1}^{n}\frac{{\left(f_{i}-f_{e}\right)}^{2}}{f_{e}}$$

*f_e_* is the expected value of the pixel point, *f_i_* is the value of the 1st-pixel point, *n* is the total number of pixels, and the significance level is 0.05. 

In addition to the Chi-square test, this paper calculates the variance of the histogram to evaluate the uniformity of encrypted image distribution. The smaller the variance is, the closer it is, the higher the consistency of the encrypted image is, the better the uniformity of the encrypted image is. In this paper, we calculate two variances of the same original image encrypted by two different sets of keys. The variance formula is as follows:

(9) $$D(z)=\frac{1}{n^{2}}\sum_{i=1}^{n}\sum_{j=1}^{n}\frac{1}{2}{\left(z_{i}-z_{j}\right)}^{2}$$

$Z=\left\{Z_{n}\right\},(n=1,2,\cdots ,256)$, Z is the frequency at which gray values occur. In the experiment, the “Lena” image is used for the experiment. The variance of the histogram of the original image is 33,860. Only one key in the two key groups is different. The variance value of the encrypted image is about 250, indicating that the average value of the number of pixels in each gray value is about 13 pixels. Experiments show that the histogram of the encrypted image is very uniform and will not provide any information to the attacker.

Table 11 shows the experimental results of the chi-square test. As can be seen from Table 11, the pixel distribution of the encrypted image follows a uniform distribution, and it is difficult for the attacker to crack the algorithm by analyzing the histogram of the encrypted image. The encrypted image will not provide any useful information to the attacker. The encryption algorithm proposed in this paper can effectively protect images from statistical attacks.

#### 5.3.2. Correlation Analysis

Adjacent pixels of the original image has a strong correlation in all directions. Only when the correlation coefficient of adjacent pixels of the encrypted image is low enough, the image processed by the encryption algorithm resist statistical attacks. Adjacent pixels are randomly selected from each direction of the original image, and the encrypted image, correlation coefficients are calculated. The correlation between adjacent pixels in the original image and the encrypted image is analyzed. The calculation formula of the correlation coefficient *r*_*xy*_ is shown in formula (10):

(10) $$r_{xy}=\frac{\mathrm{cov}(x,y)}{\sqrt{D(x)D(y)}}$$

(11) $$\mathrm{cov}(x,y)=\frac{1}{N}\sum_{i=1}^{N}\left(x_{i}-E(x))(y_{i}-E(y)\right)$$

(12) $$E(x)=\frac{1}{N}\sum_{i=1}^{N}x_{i}$$

(13) $$D(x)=\frac{1}{N}\sum_{i=1}^{N}{\left(x_{i}-E(x)\right)}^{2}$$

In the above formula, *N* is the total number of pixel points, *x* and *y* are gray values of adjacent pixels, *E*(*x*) is the average value of the pixel, *D*(*x*) is the variance, *cov*(*x*, *y*) is the correlation function, and *r*_*xy*_ is the correlation coefficient, the higher the absolute value, the stronger the correlation.

Table 12 shows the pixel correlation coefficients of the original image and the encrypted image in all directions. The correlation coefficients of adjacent pixels in the original image are all close to 1, and the correlation coefficients of encrypted images are all close to 0, which indicates that the original image has a significant correlation between pixels in different directions. Still, the correlation of adjacent pixels is eliminated after encryption algorithm processing.

**Figure 5:**
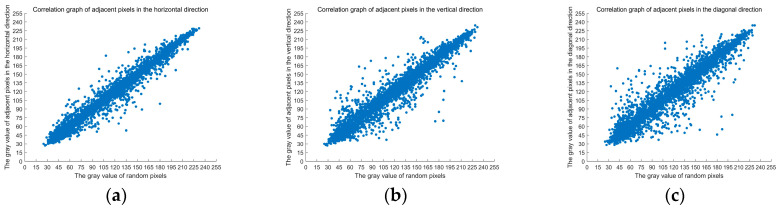

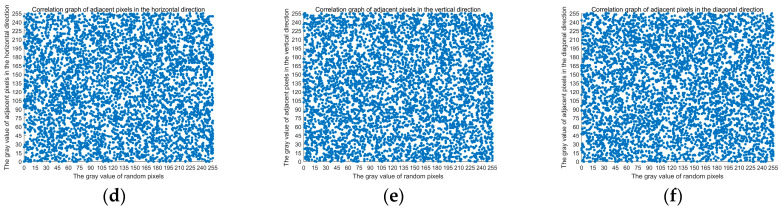
Correlation between adjacent pixels of the "Lena" image before and after encryption.

To more intuitively compare the correlation between adjacent position data values of images before and after encryption, the image “Lena” and its encrypted images are taken as examples. The correlation of their two adjacent pixels in the horizontal, vertical, and diagonal directions are plotted respectively, the abscissa is the data value of the random point position, and the ordinate is the data value of the random point adjacent position, as shown in Figure 5.

In Figure 5a is the horizontal distribution of adjacent pixels before encryption, Figure 5b is the vertical distribution of adjacent pixels before encryption, Figure 5c is the diagonal distribution of adjacent pixels before encryption, Figure 5d is the horizontal distribution of adjacent pixels after encryption, Figure 5e is the vertical distribution of adjacent pixels after encryption, and Figure 5f is the diagonal distribution of adjacent pixels after encryption.

As can be seen from Figure 5, the adjacent pixel points of the original image are continuously distributed, and the adjacent pixel point values of the ciphertext image are randomly distributed. They are distributed all over the two-dimensional space. The ciphertext image eliminates the correlation of the adjacent pixels and masks the data characteristics of the original image.

The experimental results show that the encryption algorithm greatly reduces the pixel correlation of encrypted images, and attackers cannot obtain useful information from encrypted images through statistical attacks. The algorithm in this paper has high security, and statistical attacks cannot crack the encryption algorithm in this paper.

#### 5.3.3. Information Entropy Analysis

There are many indexes to judge the randomness of pixels, of which the information entropy is the most commonly used and essential index, and its specific mathematical definition is shown in Equation (14):

(14) $$H(x)=-\sum_{i=0}^{2^{N}-1}p(x_{i})\log_{2}p(x_{i})$$

Among them, the proportion of image gray value *x*_*i*_ is expressed by (*x*_*i*_), and the gray level of the image is 2^*N*^. If the gray level of the image is *M*, then *H*_*max*_ = log_2_*M* (bit/symbol) has its maximum entropy. When *M* = 256 = 2^8^, *H*_*max*_ = 8, the closer the number is to 8, the less likely the attacker is to crack the encrypted image. In the experiment, information entropy was calculated for the ciphertext images of ten test images, comparison with the literature [51], and the results are shown in Table 13.

As can be seen from Table 13, the Shannon entropy of the encrypted image exceeds 7.99, which is very close to the theoretical value of 8, and the entropy value is higher than the literature [51]. Therefore, the encrypted image generated by the encryption algorithm proposed in this paper has good randomness and sufficient security to resist statistical attacks.

### 5.4. Analysis of Known-Plaintext and Selective-Plaintext Attacks

In the encryption process, the algorithm uses the SHA-256 function, and the key space includes the hash value of the original image, so the diffusion and scrambling process is closely related to the original image. The algorithm is susceptible to slight changes in the original image.

**Figure 6:**
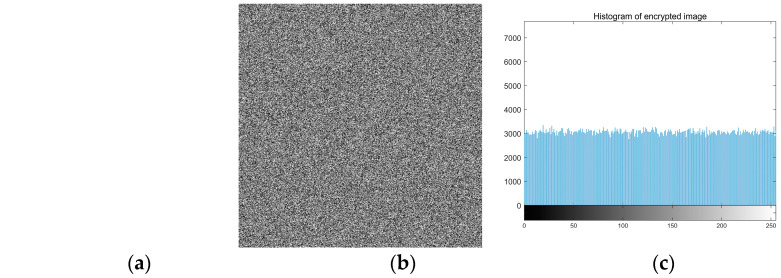

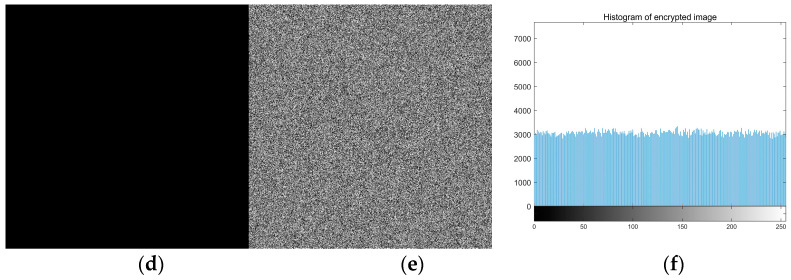
Experimental results of all-white and all-black images; (**a**) All-white image; (**b**) Encrypt image; (**c**) Encrypted image histogram; (**d**) All-black image; (**e**) Encrypt image; and (**f**) Encrypted image histogram.

In this paper, we test all black and all white images to analyze whether the experiment will fail the encryption algorithm. The chi-square test results of information entropy, NPCR, UACI, pixel correlation coefficient, and histogram are shown in Table 14. Figure 6 shows the histogram of the original image and encrypted image. The unified average change intensity (UACI) is one of the important analyses of the sensitivity tests. The number of pixel changing rate (NPCR) manifests the possibility of the differential attack by its sensitivity. The typical values of NPCR and UACI are 99.61% and 33.46%, respectively. The calculation formula is as follows:

(15) $$UACI=\frac{\sum_{i=1}^{M}\sum_{j=1}^{N}\left|P_{1}(i,j)-P_{2}(i,j)\right|}{255\times M\times N}\times 100\%$$

(16) $$NPCR=\frac{\sum_{i=1}^{M}\sum_{j=1}^{N}Q(i,j)}{M\times N}\times 100\%$$

(17) $$Q(i,j)=\left\{ \begin{array}{l} 0,P_{1}(i,j)=P_{2}(i,j)\\ 1,P_{1}(i,j)\neq P_{2}(i,j) \end{array}\right.$$

Table 14 shows that the entropy of encrypted images is more significant than 7.99. The UACI value and the NPCR value approach the theory, the results show that the two images before and after encryption, are entirely different; The correlation coefficients of pixels in three directions are close to 0 and accord with uniform distribution, so useful information cannot be obtained. This algorithm can resist known plaintext attacks and selective plaintext attacks. The experimental results show that the encryption algorithm in this paper cannot be cracked by using all-white and all-black images.

### 5.5. Differential Attack Analysis

Take the image “5.2.08” as an example [52], compare the image encryption algorithm in this paper with that in literature [53,54], and obtain the average value of UACI and NPCR, as shown in Table 15. To make the encryption algorithm resist the differential attack, we must make the algorithm very sensitive to the original image, and the small changes of the original image can produce significant changes in the encrypted image. There are many evaluation criteria for the anti-differential cryptanalysis ability of the encryption algorithm, which are generally measured by average change intensity (UACI) and pixel change rate (NPCR). UACI and NPCR values of different images are shown in Table 16.

To more intuitively display the influence on the encrypted image when the pixel value at a certain position of the image changes, taking the image “Lena” as the experimental object, Compare the encrypted image after changing the pixel with the encrypted image of the original image, the experimental results are shown in Table 17.

From the above two tables, it can be seen that the encryption scheme is susceptible to the changes in the original image, and UACI and NPCR are close to the theoretical values. Even if the changes in the original image are minimal, two completely different encrypted images can be obtained. Therefore, the algorithm in this paper can effectively resist differential attack.

### 5.6. Analysis of noise attack

In fact, in the process of image transmission, it is often affected and destroyed by noise, resulting in inevitable errors, which makes it difficult to decrypt. To test the anti-noise performance of the algorithm, different levels of Gaussian noise and salt and pepper noise are added to the ciphertext image to simulate the noise in the transmission process, which is a reasonable assumption derived from the real physical channel. If the encryption system is sensitive to noise, the change of the encrypted image will hinder the image decryption [42]. In Lena’s encrypted image, Gaussian noise with different variance, salt and pepper noise with different intensity are added respectively, and the noise is evenly distributed in the encrypted image.

**Figure 7:**
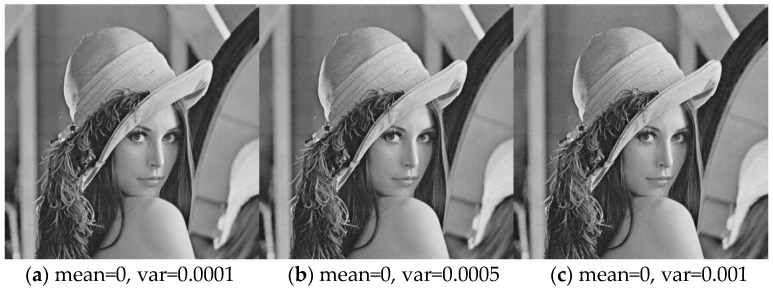

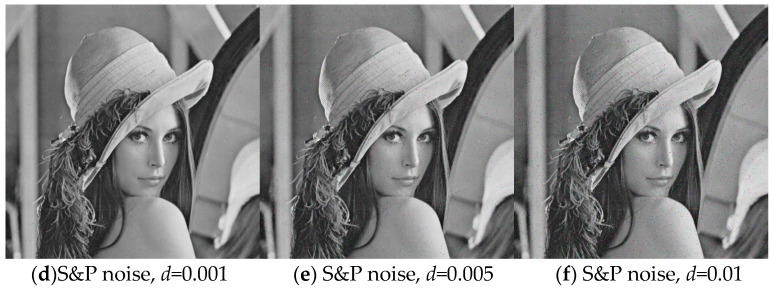
(**a**–**c**) Decrypted image with Gaussian noise; (**d**–**f**) Decrypted image with Salt and pepper noise.

Figure 7 is a decrypted image in each case. For each image in Figure 7, the correlation coefficient between the decrypted image and the noisy decrypted image is determined by the structural similarity method. The closer the structural similarity coefficient i

s to 1, it is proved that the smaller the error between the decrypted image and the noisy decrypted image is, the stronger the anti-noise ability of the system is. As shown in Table 18, the measurement value of the correlation coefficient between two images proves the robustness of noise. The correlation coefficient is close to 1 and larger than the literature [47]. That is to say, and the decrypted image still retains the whole information of the original image, which verifies the system’s ability of anti-noise attack.

### 5.7. Analysis of Algorithm Complexity and Performance

In addition to paying attention to security, the encryption algorithm should also consider the operation speed of the algorithm, usually including time complexity and space complexity. The algorithm with low complexity has fast processing speed and can be used for real-time encryption.

The time complexity of the algorithm in this paper depends on key stream generation, permutation operation, and diffusion operation. Let the size of the original image P be *m* × *n*. The length of chaotic sequences generated by the system is *m* × *n*, and the time complexity is O(*m* × *n*). The diffusion part includes DNA encoding and XOR, with a complexity of O(*m* × *n*).

Table 19 shows that compared with the algorithms of the References [55,56,57,58,59], the encryption algorithm in this paper has lower time complexity. Spatial complexity is an important index to measure the complexity of algorithms. In this paper, the Knuth–Durstenfeld algorithm is applied to the scrambling process, where the space complexity of the algorithm is O (1), which means that the encryption algorithm does not need more complicated calculations in the scrambling phase. However, many existing encryption schemes [13,15,20] have larger space complexity than O(1) and are less efficient than the algorithm in this paper.

Taking the image “Lena” as an example, the correlation coefficients of adjacent pixels in the horizontal, vertical, and diagonal directions of the encrypted image are calculated, as shown in Table 20. The results prove that the encryption scheme in this paper can achieve the encryption effect of the encryption algorithm in reference [55,56,57,58,59]. The application of the Knuth–Durstenfeld algorithm to scrambling not only ensures the scrambling impact, but also reduces the complexity of the algorithm and improves the efficiency of the algorithm. Moreover, with the increase of image size, the advantages of the algorithm will gradually become obvious.

In Table 20, the correlation coefficient of the encrypted image of the encryption scheme in this paper is less than or close to that of documents [57,60,61,62], which indicates that the shuffling algorithm adopted in this paper can achieve the scrambling effect of other encryption algorithms. The information entropy of encrypted images is larger than that of documents [57,60,61,62] and closer to the theoretical value of 8, which proves that the images encrypted by the encryption scheme in this paper have good randomness. Combined with the above experimental results, the encryption scheme in this paper is superior to that in literature [57,60,61,62].

## 6. Conclusions

This paper proposes hybrid secure image encryption based on the shuffle algorithm and the hidden attractor chaos system. The hidden attractor chaotic system generates the chaotic sequence required for image encryption. The NIST and TESTU01 tests are carried out on the chaotic sequence generated by the hidden attractor chaotic system, which proves that the hidden attractor chaotic system is suitable for image encryption. Because the shuffling algorithm has good randomness, this paper uses a shuffling algorithm to scramble images. In this paper, the security of the scheme is verified through a large number of experimental analyses: exhaustive attack, statistical attack, differential cryptanalysis, known-plaintext attack and selective plaintext attack, and noise attack. The experimental results show that the scheme is useful and practical in the field of image encryption, but there are still many areas to be explored and improved. The algorithm in this paper is mainly designed for grayscale images, which need to convert data into grayscale images and encrypt them. In the future, the range of encryption algorithm can be expanded to encrypt more image types.

## Figures and Tables

**Table 1:** Influence of initial value and parameters on system.

Initial Value	Parameters	System State
(0.2,0.1,0.75,−2)	a=10,b=25,c=−2.5,m=1,k=1	Double-scroll hyperchaos
(0.2,0.8,0.75,−2)	a=10,b=25,c=−4.66,m=1,k=1	Chaos
(0.2,0.8,0.75,−2)	a=10,b=25,c=2,m=1,k=1	Periodic orbits
(0.2,0.1,0.75,−2)	a=10,b=25,c=−4.66,m=1,k=1	Hyper-chaos

**Table 2:** The influence of parameter change on the system.

Value Range of c	System State
(−7.45,−4.96)∪(−4.94,−4.68)∪(−4.66,−4.12)∪(−0.46,0.24)	Chaos
(−4.96,−4.94)∪(−4.68,−4.66)∪(−4.12,−0.46)∪[1.84,1.88]	Hyper-chaos
(−0.24,0.154)	Chaos or quasi-periodic orbits or Periodic orbits
[0.154,1.84)∪[1.88,2.84]	Periodic
[2.84,8.54]	Quasi-periodic
(8.54,9)	Chaos

**Table 3:** Random test results.

Randomness Test		*p*-Value	Result
Frequency test		0.756086	Pass
Block Frequency test		0.965353	Pass
Runs test		0.756043	Pass
Longest Run of One’s test		0.445124	Pass
Matrix Rank test		0.152412	Pass
Discrete Fourier Transform test		0.756312	Pass
Non-Overlapping Template Matchings test		0.232635	Pass
Overlapping Template Matchings test		0.953691	Pass
Universal test		0.970868	Pass
Linear Complexity test		0.851026	Pass
Serial test	*p*_value1	0.179212	Pass
	*p*_value2	0.432451	Pass
Approximate Entropy test		0.631205	Pass
Cumulative Sums test	Forward	0.078968	Pass
	Reverse	0.083989	Pass
Random Excursions test		0.221075	Pass
Random Excursions Variant test		0.436787	Pass

**Table 4:** Test results of TESTU01.

Test Suite	Evaluation of Data Volume	Total Tests	Test Result
**SmallCrush**	6Gb	15	Pass
**Crush**	973Gb	144	Pass
**BigCrush**	10Tb	160	Pass
**Alphabet**	953Mb	17	Pass
**Rabbit**	953Mb	40	Pass
**PseudoDIEHAR**	5Gb	126	Pass
**FIPS-140-2**	19Kb	16	Pass

**Table 5:** DNA encoding rules.

	0	1	2	3	4	5	6	7
**A**	00	00	01	01	10	10	11	11
**T**	11	11	10	10	01	01	00	00
**G**	01	10	00	11	00	11	01	10
**C**	10	01	11	00	11	00	10	01

**Table 6:** DNA XOR operation.

⊕	A	T	G	C
**A**	A	T	G	C
**T**	T	A	C	G
**G**	G	C	A	T
**C**	C	G	T	A

**Table 7:** Experimental parameters of encryption and decryption process.

Items	Value
**Parameters**	a=10,b=25,c=4.6,m=1,k=25
**Initial values**	(0.2,0.1,0.75,−2)
**256-bit key (binary format)**	1110110111010001101110111010100000001011100011100110000000000000 0000000000000000000000000000000000000000000000000000000000000000 0000000000000000000000000000000000000000000000000000000000000000 0000000000000000000000000000000000000000000000000000000000000000

**Table 8:** Key space comparison of different algorithms.

Algorithm	The Algorithm in This Paper	Ref. [43]	Ref. [44]	Ref. [45]	Ref. [46]	Ref. [47]
Key space	2^327^	2^299^	2^299^	2^256^	2^256^	2^319^

**Table 9:** Results of key sensitivity analysis experiment 1.

Image Name	Image	Encrypted Image	Error Key Decryption Image	Decryption Image
Lena	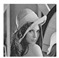	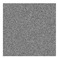	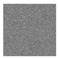	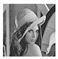
5.2.08	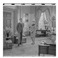	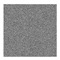	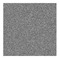	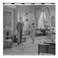
5.2.09	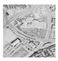	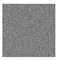	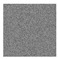	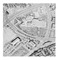
5.2.10	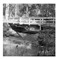	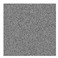	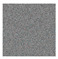	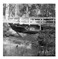
7.1.02	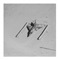	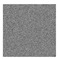	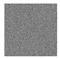	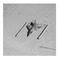
7.1.03	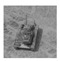	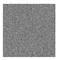	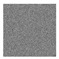	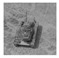
7.1.05	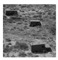	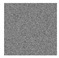	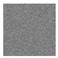	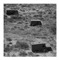
7.1.08	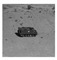	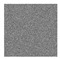	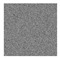	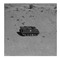
7.1.10	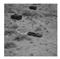	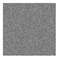	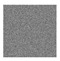	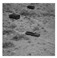
boat	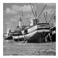	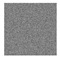	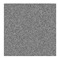	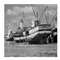

**Table 10:** Results of key sensitivity analysis experiment 2.

Image Name	Encrypted Image	Error Key Decryption Image(*x_0_* + 10^−15^)	Error Key Decryption Image(*y_0_* + 10^−15^)	Error Key Decryption Image(*z_0_* + 10^−15^)	Error Key Decryption Image(*h_0_* + 10^−15^)	Decryption Image
Lena	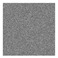	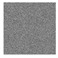	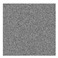	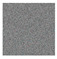	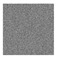	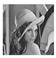
5.2.08	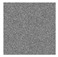	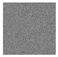	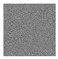	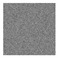	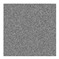	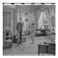

**Table 11:** Histogram of the original image and encrypted image.

Image Name	Histogram of the Original Image	Histogram of the Encrypted Image	χ2	*p*-Values
Lena	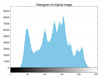	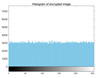	278.7992	0.7896
5.2.08	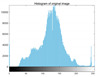	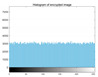	252.6531	0.5832
5.2.09	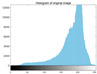	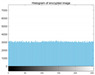	259.7123	0.6816
5.2.10	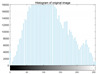	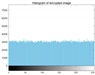	264.5217	0.7124
7.1.02	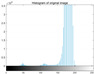	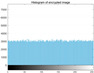	269.1632	0.6195
7.1.03	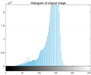	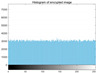	251.6328	0.5913
7.1.05	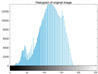	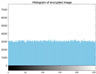	272.6374	0.7351
7.1.08	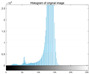	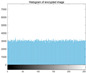	262.6891	0.6237
7.1.10	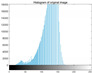	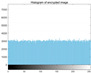	275.8627	0.5769
boat	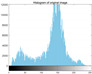	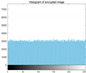	254.1951	0.6365

**Table 12:** The correlation coefficient of adjacent pixels in the image.

Image	Direction	Original Image	Encrypted Image	
			The Algorithm in This Paper	Ref. [50]
Lena	Horizontal	0.9755	−0.0045	−0.0048
	Vertical	0.9850	−0.0103	−0.0112
	Diagonal	0.9626	0.0022	−0.0045
5.2.08	Horizontal	0.9446	−0.0071	−0.0251
	Vertical	0.8856	0.0002	−0.0213
	Diagonal	0.8387	−0.0045	−0.0232
5.2.09	Horizontal	0.9077	0.0012	−0.0014
	Vertical	0.8594	−0.0023	−0.0056
	Diagonal	0.8110	0.0117	−0.0049
5.2.10	Horizontal	0.9380	−0.0093	−0.0190
	Vertical	0.9250	0.0167	−0.0182
	Diagonal	0.8910	0.0120	−0.0079
7.1.02	Horizontal	0.9338	−0.0062	−0.0002
	Vertical	0.9439	−0.0036	−0.0090
	Diagonal	0.8801	0.0193	−0.0066
7.1.03	Horizontal	0.9480	−0.0036	−0.0202
	Vertical	0.9339	−0.0173	−0.0200
	Diagonal	0.9054	0.0012	−0.0013
7.1.05	Horizontal	0.9423	0.0083	−0.0086
	Vertical	0.9089	−0.0094	−0.0103
	Diagonal	0.8926	0.0142	−0.0079
7.1.08	Horizontal	0.9572	−0.0150	−0.0195
	Vertical	0.9261	0.0002	−0.0127
	Diagonal	0.9206	0.0119	−0.0124
7.1.10	Horizontal	0.9634	0.0197	−0.0201
	Vertical	0.9483	−0.0199	0.0135
	Diagonal	0.9288	0.0169	−0.0182
boat	Horizontal	0.9415	−0.0130	−0.0100
	Vertical	0.9696	0.0111	−0.0124
	Diagonal	0.9209	−0.0182	−0.0185

**Table 13:** The result of information entropy.

Image Name	The Entropy of the Original Image	The Entropy of the Encrypted Image	Ref. [51]
Lena	7.4455	7.9983	7.9086
5.2.08	7.2010	7.9986	7.9025
5.2.09	6.9940	7.9991	7.9027
5.2.10	5.7056	7.9989	7.9022
7.1.02	4.0045	7.9992	7.8936
7.1.03	5.4957	7.9994	7.9007
7.1.05	6.5632	7.9982	7.9022
7.1.08	5.0534	7.9985	7.9024
7.1.10	5.9088	7.9993	7.9027
boat	7.1914	7.9986	7.9025

**Table 14:** Experimental results of all-white and all-black images.

Images		Full White Image	Full Black Image
**Entropies**		7.9971	7.9972
**UACI**		0.3348	0.3337
**NPCR**		0.9959	0.9960
**Correlation coefficients**	**Horizontal**	0.0051	0.0035
	**Vertical**	0.0026	0.0060
	**Diagonal**	0.0020	0.0028
**χ** **2**		263.4922	249.8672
***p*** **-values**		0.6559	0.4210

**Table 15:** Comparison of unified average change intensity (UACI) value and number of pixel changing rate (NPCR) of different algorithms.

	The Algorithm in This Paper	Ref. [53]	Ref. [54]
UACI	33.60%	33.05%	33.53%
NPCR	99.61%	99.52%	99.60%

**Table 16:** UACI and NPCR values of images.

Image Name	UACI	NPCR
Lena	33.51%	99.63%
5.2.08	33.60%	99.61%
5.2.09	33.81%	99.59%
5.2.10	33.75%	99.63%
7.1.02	33.62%	99.62%
7.1.03	33.54%	99.56%
7.1.05	33.86%	99.62%
7.1.08	33.53%	99.60%
7.1.10	33.64%	99.59%
boat	33.79%	99.58%

**Table 17:** UACI and NPCR when a pixel value changes.

Pixel Location	UACI	NPCR
**(1,1)**	33.49%	99.64%
**(511,511)**	33.48%	99.60%
**(1,511)**	33.51%	99.66%
**(511,1)**	33.49%	99.60%
**(256,256)**	33.44%	99.62%

**Table 18:** The structural similarity between decrypted image and decrypted image with noise.

		The Algorithm in This Paper	Ref. [47]
Gaussian noise	var = 0.0001	0.9518	0.9076
	var = 0.0005	0.8410	0.8266
	var = 0.001	0.7849	0.7667
S&P noise	d = 0.001	0.9975	0.9973
	d = 0.005	0.9871	0.9862
	d = 0.01	0.9720	0.9683

**Table 19:** Comparison of time complexity.

Algorithm	Encryption Process	
	Scrambling	Diffusion
Algorithm in this paper	O(m×n)	O(m×n)
Ref. [40]	O(4m×n)	O(4m×n)
Ref. [41]	O(8m×n×log(8m×n))	Same as this paper
Ref. [42]	Same as Ref. [41]	Same as this paper
Ref. [43]	O(m×n×log(m×n))+O(4m×n×log(4m×n))	O(4m×n)
Ref. [44]	O(2m×n)+O(3m×n)	Same as this paper

**Table 20:** Performance comparison of algorithms.

Algorithm	Entropy	Correlation Coefficients		
		Horizontal	Vertical	Diagonal
Algorithm in this paper	7.9983	−0.0045	−0.0103	0.0022
Ref. [55]	7.9974	−0.0230	0.0019	0.0034
Ref. [56]	-	0.0102	−0.0053	−0.0161
Ref. [57]	-	−0.0038	−0.0026	0.0017
Ref. [58]	7.9974	0.0241	-0.0194	0.0243
Ref. [59]	7.9973	0.0000	−0.0011	0.0074
Ref. [60]	7.9976	0.0030	−0.0024	−0.0034
Ref. [61]	7.9974	−0.0098	−0.0050	−0.0013
Ref. [55]	7.9974	−0.0230	0.0019	−0.0034
Ref. [62]	7.9973	−0.0226	0.0041	0.0368
